# Microsatellite and Mitochondrial COI Provide Novel Insights Into the Population Genetic Structure of White Prunicola Scale (*Pseudaulacaspis prunicola*) in China

**DOI:** 10.1002/ece3.70865

**Published:** 2025-01-20

**Authors:** Minmin Niu, Dengen Fu, Haoyang Wang, Yun Liu, Xuanxing Du, Qing Zhao, Jiufeng Wei

**Affiliations:** ^1^ College of Plant Protection Shanxi Agricultural University Jinzhong China; ^2^ Potato Industry Development Center Yulin Shaanxi Province China

**Keywords:** genetic structure, microsatellite, mitochondrial COI, population, white prunicola scale

## Abstract

The white prunicola scale *Pseudaulacaspis prunicola* (Maskell) is an important pest of fruit and ornamental plants, characterised by its wide distribution, broad host range and distinct biological traits. In this study, a comprehensive population genetic analysis of *P. prunicola* in China was conducted, focusing on genetic diversity, genetic structure, relationships among geographical populations, and population dynamics. Microsatellite molecular and mitochondrial COI markers were used to examine the genetic diversity and structure of 19 *P. prunicola* populations across 10 provinces in China. The results revealed low genetic diversity and limited gene flow among populations. A clear geographic genetic structure was identified, with the 19 populations being, divided into four distinct groups, showing a pronounced north–south distribution pattern. Significant genetic differentiation was observed between these groups, with minimal gene exchange. COI‐based diversity analyses produced results similar to those obtained from the microsatellite markers. These findings provide valuable insights into the distribution and spread of *P. prunicola* in China and may help inform the development of effective and targeted pest control strategies.

## Graphical abstract

In China, the white prunicola scale is abundant. In this study, a detailed population genetic analysis of white prunicola scale in China was performed, including analyses of genetic diversity, genetic structure, relationships among geographical populations, and population dynamics.

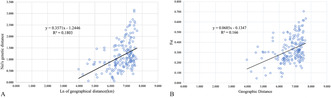

## 1 Introduction

The white prunicola scale (*Pseudaulacaspis prunicola* (Maskell 1895)) is a significant pest that primarily affects *Prunus* in temperate regions (Tang 1986; Miller and Davidson 2005). This pest can feed on over 26 genera belonging to 18 families and is currently found in seven countries or regions worldwide (García Morales et al. 2016). It has caused notable damage to Japanese plum ( *Prunus salicina* ) in Hawaii (Maskell 1895) and cherry trees in Washington D.C. (García Morales et al. 2016). In China, *P. prunicola* has spread widely through the transport of seedlings and trade, often leading to wilting and even death of host plants.

*Pseudaulacaspis prunicola* has often been confused with *P. pentagona* (Targioni Tozzetti, 1886) and *P. simplex* Takagi, 1961. Most of the reports likely refer to *P. pentagona* , as these studies typically focus on warmer areas and hosts other than *Prunus* (García Morales et al. 2016). The only confirmed account of the life history of *P. prunicola* was provided by Stimmel in 1982 (García Morales et al. 2016). Stimmel (1982) documented the seasonal history of the species on *Prunus serrulata* Lindl. (Japanese flowering cherry) in northeastern Pennsylvania. Miller and Davidson (2005) observed *P. prunicola* in College Park, Maryland in 1981 on *P. serrulata* , where it produced three generations per year: in early May, early July and late August (Miller and Davidson 2005). Only mated adult females overwintered (Miller and Davidson 2005).

Population genetic structure is shaped by various factors, including the dispersal potential of species (Francoso et al. 2016; Portnoy et al. 2015), geographical barriers (Hirao, Kubota, and Murakami 2015; Ye et al. 2016), historical processes (Campos et al. 2013; Hewitt 2000) and ecological conditions (Katz, Taylor, and Davis 2018). Multiple molecular markers, such as single‐copy nuclear gene sequences, internal transcriptional spacer regions, single nucleotide polymorphisms, microsatellite loci, and mitochondrial and chloroplast DNA, have been employed in systematics and biogeography research (Card et al. 2016; Garrick et al. 2015; Zhou et al. 2021).

Microsatellites, also known as simple sequence repeats (SSR), are typically composed of 1–6 repeats of mono‐ to hexa‐nucleotide motifs, which are non‐randomly distributed across coding and non‐coding regions of the genome (Zane, Bargelloni, and Patarnello 2002). SSR markers are characterised by their ability to amplify across species, high repeatability, co‐dominant inheritance, rich polymorphism information and easy operation. They are widely applied in molecular breeding, population genetic analyses, the identification of closely related species, gene mapping and cloning, and the construction of genetic linkage maps (van Zonneveld et al. 2012; Tang, Tao, and Du 2015; Zhu et al. 2019). Du et al. (2023) successfully assembled transcriptome data for *P. prunicola* collected from Shanxi, Taigu in China and developed 29 microsatellite primers, providing a molecular resource for future population genetics and invasion biology studies.

Similarly, the mitochondrial genome offers several advantages, including its simple structure, maternal inheritance, rapid evolutionary rate and low recombination (Cann, Rickards, and Lum 1994; Wang et al. 2019). Mitochondrial COI genes are easily amplified by universal primers, ensuring sufficient variation with minimal insertions and deletions, and provide strong phylogenetic signals (Hebert et al. 2003). Lu et al. (2023) used morphological characteristics, molecular evidence (COI sequence data) and ecological niches to distinguish between *P. pentagona* and *P. prunicola*.

Because single‐locus datasets are insufficient to fully represent the evolutionary history of a species, an increasing number of systematic geographic studies are combining multiple molecular markers (Lu et al. 2016; Myers et al. 2013; Zhou et al. 2021). In this study, we analyse the genetic structure and diversity of different geographical populations of *P. prunicola* in China using microsatellite and COI molecular markers. The objectives of the study are as follows: (1) to assess genetic diversity and population structure of *P. prunicola* in China using SSR and mitochondrial COI data, (2) to explore relationships between different geographical populations of *P. prunicola* and (3) to infer the historical dynamics of the species.

## 2 Material and Methods

### 2.1 Sample Collection

Samples of *P. prunicola* were collected over a wide range of regions in North, South, Central, East and Northwest China. The host plants all belonged to the family Rosaceae. Detailed collection information is shown in Table 1. The specimens and their host plant tissues were stored at −20°C for further molecular experiments.

**TABLE 1:** Sample collection information for *P. prunicola*.

Location	Population	Host plant	Date	Lon/Lat	Number 1	Number 2
Yinchuan, Ningxia	NXYC	*Amygdalus persica*	2021/10	106.26/38.47	14	20
Qingtongxia, Ningxia	NXQTX	*Amygdalus persica*	2021/10	106.08/38.02	12	20
Zhongwei, Ningxia	NXZW	*Amygdalus persica*	2021/10	105.19/37.50	12	20
Dingbian, Shaanxi	SNDB	*Amygdalus persica*	2021/08	107.50/37.58	8	8
Yangling, Shaanxi	SNYL	*Amygdalus persica*	2021/09	108.07/34.30	12	20
Linfen, Shanxi	SXLF	*Amygdalus persica*	2021/06	111.48/36.08	12	19
Taiyuan, Shanxi	SXTY	*Amygdalus persica*	2021/08	112.53/37.98	12	20
Taigu, Shanxi	SXTG	*Amygdalus persica*	2021/04	112.55/37.35	12	18
Yangquan, Shanxi	SXYQ	*Amygdalus persica*	2021/09	113.57/37.86	12	20
Leishan, Guizhou	GZLS	*Amygdalus persica*	2021/08	108.09/26.37	12	20
Guilin, Guangxi	GXGL	*Cerasus jamasakura*	2021/07	110.67/25.61	12	20
Fangchenggang, Guangxi	GXFCG	*Cerasus jamasakura*	2021/07	108.34/21.62	12	20
Jishou, Hunan	HNJS	*Amygdalus persica*	2018/02	109.72/28.29	12	20
Hanzhong, Shaanxi	SNHZ	*Amygdalus persica*	2014/07	107.02/33.07	12	20
Zhangjiakou, Hebei	HBZJK	*Amygdalus persica*	2021/07	115.28/39.96	12	16
Longyan, Fujian	FJLY	*Amygdalus persica*	2017/04	117.00/25.09	9	9
Kunming, Yunnan	YNKM	*Amygdalus persica*	2021/07	102.68/24.88	12	13
Quanzhou, Fujian	FJQZ	*Amygdalus persica*	2022/07	118.58/24.93	10	10
Quzhou, Zhejiang	ZJQZ	*Amygdalus persica*	2022/07	118.94/28.98	12	16

*Note:* Number 1 represents the number of samples used in microsatellite analyses, and Number 2 represents the number of samples used in mitochondrial COI analyses.

### 2.2 DNA Preparation and PCR Amplification

The total genomic DNA of the specimens was extracted using the Ezup Column‐based Animal Genomic DNA Extraction Kit (Shanghai Bioengineering Co. Ltd., Shanghai, China). Genomic DNA samples were used for PCR to amplify an approximately 650 base pair (bp) fragment of the *COI* barcode region using the universal primers PcoF1 (5′‐CCTTCAACTAATCATAAAAATATYAG‐3′) (Park et al. 2010) and LepR1 (5′‐TAAACTTCTGGATGTCCAAAAAATCA‐3′) (Park et al. 2011). The 30 μL amplification cocktail contained 9.5 μL of ddH_2_O, 13.5 μL of 2× Taq Master mix (1 mL), 1.5 μL of each primer (10 μM) and 4 μL of template DNA. The PCR was conducted under the following conditions: initial denaturation at 95°C for 3 min; followed by 5 cycles of 95°C for 1 min, 48°C for 2 min and 72°C for 1 min and 35 cycles of 95°C for 1 min, 51°C for 2 min and 72°C for 1 min; and a final extension at 72°C for 8 min. The PCR products were visualised using 1% agarose gel electrophoresis and forward sequenced by Qingke Biotechnology Co. Ltd. (Shaanxi, China).

### 2.3 Synthesis and PCR Amplification of Microsatellite Fluorescent Labelled Primers

Polyacrylamide gel electrophoresis was used for 29 pairs of microsatellite primers screened in our previous study (Du et al. 2023), and nine pairs with highly polymorphic bands were selected for genetic diversity analyses of *P. prunicola* (Table 2). The fluorescently labelled primers were based on Schuelke (2000), with an additional M13 (21) universal primer (5′‐TGTAAAACGAGCGGCCAGT‐3′), and three primers were used for amplification. The first primer was the microsatellite site forward primer M13‐F with the M13 (21) universal primer added to the 5′ end, the second primer was the microsatellite site reverse primer R and the third primer was 5′ M13 (21) universal primer FAM‐M13 (FAM‐5′‐TGTAAAACGACGGCCAGT‐3′) with carboxyfluorescein (FAM) at the end. Each primer was synthesised by Sangon Biotechnology Co. Ltd. (Shanghai).

**TABLE 2:** SSR loci and primer sequences in *P. prunicola*.

Locus name	Motif	Forward primer	Reverse primer	Annealing temperature (°C)
PP_10	(CATT)7	ATGGATTCGACGCATGTGGA	GCCAAGCGCCATCTTTGAAA	59
PP_12	(TGTA)12	AATCTGCAGACGGACCTTGG	AAACAAGCGTACAGAAGCGC	60
PP_24	(GTC)6	GTCACAGTCGGACCGAAACT	GGGTAGTCTATTTGGCGCGA	59
PP_30	(TTA)5	ACGAATAAACACCACTGCGC	AACAAACCGCGCATGTATGG	59
PP_37	(GAA)14	TGTCACTACCCGCGTTCAAA	ACGTTTCGTCTTCGTCGTCA	59
PP_49	(TAA)7	GTCTTAACCCGAGCACCGAT	TGCTGTTCTGATCGACGGAC	59
PP_50	(ACA)10	CGGTAACTGTTGGTCCTGGT	ATGCTGACGCCTTTCTTTGC	59
PP_25	(CGC)9	TCACCTCGTTCATCTGCACC	ACGTCTGCGAGAATCGTCTC	60
PP_46	(CTG)12	CGGTGGAGCTTGGTTCTGAT	CATCCTCAACAAGGCGCCTA	60

Amplification was carried out using two rounds of PCR, with the first‐round amplification product used as the template for the second round. The first‐round PCR system was 20 μL: DNA Template 1 μL (4 ng), 2× TaqMasterMix 10 μL, primer (M13‐F) 0.5 μL, primer (R) 0.5 μL, ddH_2_O to 20 μL. The reaction conditions were as follows: pre‐denaturation at 94°C for 5 min; 94°C denaturation for 30 s, annealing temperature (Table 2) for 30 s, 72°C extension for 30 s, a total of 30 cycles; extension at 72°C for 10 min and storage at 4°C. The second‐round PCR system was 20 μL: DNA Template (previous round of amplification product) 1 μL, 2× TaqMasterMix 10 μL, primer (FAM‐M13) 0.5 μL, primer (R) 0.5 μL, ddH_2_O replenishment to 20 μL. The reaction conditions were as follows: pre‐denaturation at 94°C for 5 min; 94°C denaturation for 30 s, 53°C annealing for 30 s, 72°C extension for 30 s, a total of 12 cycles, extension at 72°C for 10 min and storage at 4°C. Finally, PCR amplification results were detected by 1% agarose gel electrophoresis at 110 V and 20 min.

The PCR amplification product containing the target band was sent to Qingke Biotechnology Co. Ltd. (Kunming) for capillary electrophoresis detection using the ABI 3730xl DNA Analyser (Applied Biosystems). Genotyping was performed using GeneMapper 4.0 (Applied Biosystems). A second amplification was performed for sites or individuals that were not successfully amplified in the first amplification, with all other conditions remaining unchanged except for the appropriate adjustment of the annealing temperature.

Basic genetic diversity parameters, including the average number of alleles (*N*_*a*_), effective number of alleles (*N*_*e*_), Shannon–Wiener index (*I*), observed heterozygosity (*H*_*o*_), expected heterozygosity (*H*_*e*_), unbiased expected heterozygosity (*uH*_*e*_), inbreeding coefficient (*F*_IS_), overall inbreeding coefficient (*F*_IT_), population genetic differentiation coefficient (*F*_ST_), private alleles richness (*P*_*a*_), allele frequencies by population over loci, and the pairwise matrix of Nei's unbiased genetic distances were calculated using GenAlEx 6.5 (Peakall and Smouse 2012) in Excel. Linkage disequilibrium (LD) and Hardy–Weinberg Equilibrium (HWE, *p* < 0.0056) were evaluated using GenePop'007 (Rousset 2008) by applying the Markov chain method. The dememorisation number, number of batches and number of iterations per batch were set to 10,000, 5000 and 10,000, respectively. FreeNA (Chapuis and Estoup 2007) was used to calculate the null allele frequency of each locus. The gene diversity (*D*), number of alleles (*N*) and allelic richness (*A*_*r*_) (after adjusting for sample size) were calculated using FSTAT 2.9.4 (Goudet 2003). The polymorphism information content (PIC) was calculated using PowerMarker 3.25 (Liu and Muse 2005).

### 2.4 Analysis of Population Genetic Structure

*F*‐statistics and gene flow (*N*_*m*_) as well as Ewens‐Watson neutrality test (with 1000 repetitions) were performed for all loci using POPGENE 1.32 (Yeh, Yang, and Boyle 2000).

According to the Nei's unbiased standard genetic distance matrix calculated using GenAlEx, a cluster analysis was performed using the unweighted pair‐group method with arithmetic means (UPGMA) and neighbour‐joining method (NJ) in the neighbour software package of PHYLIP V3.698 (Felsenstein 2019). The clustering results were visualised using the ITOL (Letunic and Bork 2024) online website.

Population structure was evaluated using Structure 2.3.4. (Falush, Stephens, and Pritchard 2003). This software uses a mixed ancestry model without any population information to determine the number of groups. *K* values of 1–10 were evaluated, the total number of Markov chain repeats (MCMC) was 1,000,000 and the burn‐in was 100,000; each *K* value was iterated 20 times. Structure Harvester (Earl and Vonholdt 2012) was then used to determine the optimal *K* value based on Ln *Pr*(*X*|*K*) and Δ*K* values. However, when the maximum Δ*K* value did not fully represent individual differences, the grouping results at the second peak were considered for analysis. After the clustering analysis, 20 iterations with the most suitable *K* value were used for a repeated sampling analysis using CLUMPP l.1.2 (Jakobsson and Rosenberg 2007). Finally, Distruct 1.1 (Rosenberg 2004) was used to process the data and draw a stacked graph to determine the class group to which each population belongs. Arlequin 3.5.2.2 (Excoffier and Lischer 2010) was used for an analysis of molecular variance (AMOVA) with three levels of variation, including inter‐group variation, inter‐population variation and individual variation within a population, based on the number of clusters *K*. The significance level of the genetic composition was determined by repeating the analysis 1000 times.

Using GenAlEx, a principal co‐coordinates analysis (PCoA) was performed on different geographic populations of the *P. prunicola* based on the Nei's genetic distance matrix between various populations, and a two‐dimensional coordinate graph was used to display the genetic distance relationship between populations. Using GenAlEx for Mantel test, calculate the correlation coefficient between Nei's genetic distance, the natural logarithm of the *F*_ST_ and the geographic distance (km) between collection points, and analyse the correlation between genetic distance and geographic distance between paired populations.

Based on three distribution models, three testing methods can be applied. The sign test has low statistical power. The standard differences test is generally applicable to data with more than 20 polymorphic loci. The Wilcoxon signed rank test can be applied to small number of loci and has high power; it is appropriate for at least four polymorphic loci and any number of individuals and achieves good test results when there are 15–40 samples and 10–15 polymorphic loci. Bottleneck 1.2.02 (Cornuet and Luikart 1996; Piry, Luikart, and Cornuet 1999) was used to detect bottleneck effects using the Wilcoxon sign‐rank test and mode‐shift indicator test, with a repetition rate of 1000. Three mutation models were used, including the infinite allele model (IAM), stepwise mutation model (SMM) and two‐phase model (TPM), to detect genetic bottlenecks. Previous studies have shown that microsatellite data are generally better suited for TPM models than IAM and SMM models (Di Rienzo et al. 1994). Therefore, for analyses of nine polymorphic loci (Table 2) in 19 populations of *P. prunicola*, the TPM model results using Wilcoxon sign rank test were considered most accurate.

### 2.5 Genetic Diversity Analysis and Neutrality Testing

MEGA‐X 10.1.1 (Kumar et al. 2018) was used to proofread and splice the original mitochondrial COI sequence, and multi‐sequence alignment was generated using the ClustalW method for analyses of base composition and sequence characteristics. A BLAST similarity analysis of the spliced sequences was performed using the NCBI database.

To visualise the genetic relationships among haplotypes, MEGA‐X was used to calculate the genetic distances between haplotypes based on the Kimura 2‐parameter (K2P) model. The NJ method was used to construct a phylogenetic tree for a cluster analysis. PopART 1.7 (Leigh and Bryant 2015) was used to construct haplotype adjacency network diagrams for different geographic populations of *P. prunicola* and to analyse the relationships between haplotypes using the median joining method. BAPS 6.0 (Corander and Marttinen 2006) was used to perform spatial clustering of different geographic populations of *P. prunicola*.

Using spatial clustering, analysis of molecular variance (AMOVA) was performed using Arlequin 3.5.2.2. Tajima's *D* and Fu's *Fs* tests were used to evaluate different geographic populations of *P. prunicola* using Arlequin 3.5.2.2, and the population dynamics were analysed based on the sum of the squares of deviations (*SSD*) and Harpending's raggedness index (*R*).

DnaSP 6.12.03 (Rozas et al. 2017) was used to calculate genetic diversity indices, such as number of haplotypes (*H*), haplotype diversity (*H*_*d*_), nucleotide diversity (*π*) and average number of nucleotide differences (*K*). This software was also used to conduct a mismatch distribution analysis of the *COI* gene of *P. prunicola*.

## 3 Results

### 3.1 Genetic Diversity at Different SSR Loci

The Ewens‐Watterson neutrality test was used to evaluate nine microsatellite loci in 19 geographical populations of *P. prunicola*, and the results are shown in Table 3. The observed *F*‐value for one site, PP_46, was not within the 95% lower confidence limit, consistent with non‐neutral site evolution, suggesting that the site was affected by selection. The observed *F*‐values for the other eight sites were all within the confidence interval, consistent with neutral evolution without selection.

**TABLE 3:** Overall Ewens‐Watterson test results for *P. prunicola*.

Locus	Obs. *F*	Min *F*	Max *F*	Mean^a^	SE^a^	L95^a^	U95^a^
PP_10	0.4015	0.1111	0.9645	0.3726	0.0197	0.1947	0.7314
PP_12	0.2869	0.125	0.9688	0.4243	0.0248	0.2126	0.8235
PP_24	0.3519	0.125	0.9688	0.4165	0.0234	0.2157	0.8013
PP_30	0.2415	0.0625	0.9344	0.2329	0.0081	0.1266	0.4786
PP_37	0.0972	0.0455	0.9095	0.1691	0.0037	0.0957	0.3278
PP_49	0.1834	0.0625	0.9344	0.2325	0.0075	0.1242	0.4652
PP_50	0.2475	0.0909	0.9558	0.3281	0.0152	0.1618	0.6346
PP_25	0.2238	0.0909	0.9558	0.3295	0.0161	0.1671	0.6566
PP_46	0.1555	0.0909	0.9558	0.3314	0.0165	0.1678	0.6515

Abbreviations: L95, U95, upper and lower limits of 95% confidence intervals; Obs. *F*, observed set of allele frequencies squared; SE, standard error of observed *F* calculated from 1000 simulated samples.

^a^ Calculated using 1000 simulated samples.

Polymorphisms in microsatellite loci of *P. prunicola* are shown in Table 4. A total of 112 alleles were detected at nine microsatellite loci in 19 populations, with an average of 12.4 alleles per locus and the number of alleles ranges from 9 to 22. The effective number of alleles (*N*_*e*_) was 1.532–2.493; the number of effective alleles was highest at site PP_37 and lowest at site PP_12. The lowest expected heterozygosity (*H*_*e*_) was 0.255, and the lowest observed heterozygosity (*H*_*o*_) was 0.230. The highest Shannon index (*I*) of each site was 0.977, and the lowest was 0.421. *H*_*o*_ was 0.230–0.541, with an average value of 0.382, and *H*_*e*_ was 0.244–0.512, with an average value of 0.385. *Ho* for some sites was lower than *H*_*e*_, while *H*_*o*_ was higher than *H*_*e*_ at other sites. For co‐dominant genes, PIC < 0.25 indicates a small amount of genetic information and low polymorphism; 0.25 < PIC < 0.5 indicates that a site has a certain amount of genetic information and is moderately polymorphic; PIC > 0.5 indicates that a site has a large amount of genetic information and is highly polymorphic (Botstein et al. 1980). In this study, the PIC values were > 0.5, indicating that the loci were highly polymorphic and can provide sufficient genetic information. Sites PP_30, PP_37 and PP_49 deviated from HWE significantly.

**TABLE 4:** Genetic diversity at nine SSR loci of *P. prunicola*.

Locus	*N*	*N*_*e*_	*I*	*H*_*o*_	*H*_*e*_	*uH*_*e*_	PIC	HWE
PP_10	9	1.727	0.589	0.409	0.351	0.368	0.560	0.7288
PP_12	8	1.532	0.421	0.230	0.244	0.255	0.680	0.9050
PP_24	8	1.841	0.617	0.397	0.368	0.385	0.625	0.4949
PP_30	16	1.729	0.560	0.244	0.317	0.331	0.724	0.0001^a^
PP_37	22	2.493	0.977	0.477	0.512	0.536	0.896	0.0065^a^
PP_49	16	2.357	0.881	0.419	0.467	0.489	0.798	0.0000^a^
PP_50	11	1.928	0.671	0.381	0.381	0.398	0.719	0.2048
PP_25	11	2.176	0.841	0.541	0.476	0.499	0.742	0.8367
PP_46	11	1.847	0.610	0.341	0.351	0.367	0.831	0.4047

Abbreviations: *H*_*e*_, expected heterozygosity; *H*_*o*_, observed heterozygosity; HWE, Hardy–Weinberg Equilibrium; *I*, Shannon–Wiener index; *N*, number of alleles; *N*_*e*_, effective number of alleles; PIC, polymorphism information content; *uH*_*e*_, unbiased expected heterozygosity.

^a^ Indicates a significant deviation from Hardy–Weinberg equilibrium after Bonferroni correction.

The *F*‐statistics and gene flow (*N*_*m*_) of the nine microsatellite loci in this study are shown in Table 5. The range of *F*_IS_ values was −0.1355 to 0.2301, and the *F*_IS_ values for sites PP_10, PP_24 and PP_25 were negative, indicating an excess of heterozygotes. The average *F*_IS_ value was 0.0088, indicating a loss of heterozygosity at each locus. The *F*_IT_ value ranged from 0.3071 to 0.6774, which can also indicate a loss of heterozygosity at several microsatellite loci. The *F*_ST_ values ranged from 0.3898 to 0.6584 (all > 0.25), indicating that various groups of *P. prunicola* exhibit substantial genetic differentiation. The range of *N*_*m*_ values was 0.1297 to 0.3913, indicating a lack of gene exchange among groups of *P. prunicola* and obvious genetic differentiation.

**TABLE 5:** *F*‐statistics for nine SSR loci among populations of *P. prunicola*.

Locus	*F*_IS_	*F*_IT_	*F*_ST_	*N*_*m*_ ^a^
PP_10	−0.1628	0.3239	0.4186	0.3473
PP_12	0.0556	0.6774	0.6584	0.1297
PP_24	−0.0787	0.3855	0.4303	0.3310
PP_30	0.2310	0.6766	0.5795	0.1814
PP_37	0.0685	0.4707	0.4318	0.3290
PP_49	0.1031	0.4877	0.4287	0.3331
PP_50	0.0000	0.4945	0.4945	0.2555
PP_25	−0.1355	0.3071	0.3898	0.3913
PP_46	0.0285	0.5959	0.5841	0.1780
Mean	0.0088	0.4958	0.4913	0.2588

^a^ *N*_m_ = 1/4(1−*F*_ST_)/*F*_ST_.

### 3.2 Genetic Diversity Analysis of Different Geographic Populations at Nine Microsatellite Loci

FreeNA software was used to detect null alleles in the genotyping results for 221 samples of *P. prunicola* from 19 populations at nine microsatellite loci. Only a few loci had invalid alleles in a few populations. The average invalid allele frequency for each locus was low and did not affect subsequent genetic analyses.

Only a few populations showed significant deviation from HWE at a few loci. Populations in Yangquan of Shanxi Province (SXYQ), Yangling of Shaanxi Province (SNYL), Yinchuan of Ningxia Province (NXYC) and Quzhou of Zhejiang Province (ZJQZ) showed significant deviations from HWE at site PP_49, populations in Jishou of Hunan Province (HNJS) and Kunming of Yunnan Province (YNKM) showed deviations from HWE at site PP_37, and populations in NXYC and ZJQZ deviated from HWE at site PP_30. The Longyan of Fujian Province (FJLY) population deviated from HWE at site PP_24. Some populations have only one genotype at some sites, without polymorphism, with significant deviations from HWE. Among the microsatellite loci, no loci showed deviations from HWE in all populations, and no population showed deviations from HWE at all loci. The results of an LD analysis were similar to those of HWE detection. Linkage was detected in a few loci in a few populations, and there was no obvious linkage between loci.

In the HWE test, after correction by the Bonferroni continuous correction method, values of *p* < 0.0056 indicate a deviation from HWE. The HWE test was conducted on all populations, revealing significant deviations from HWE in the populations in NXYC and ZJQZ (Table 6).

**TABLE 6:** Genetic diversity in 19 populations of *P. prunicola*.

Population	*N*_*a*_	*N*_*e*_	*P*_*a*_	*A*_*r*_	*D*	*H*_*e*_	*uH*_*e*_	*H*_*o*_	*I*	*F*_IS_	HWE
SXTG	3.889	2.178	1	3.420	0.523	0.499	0.521	0.481	0.917	0.087	0.3583
SXLF	3.667	2.574	1	3.383	0.620	0.596	0.622	0.657	1.041	−0.112	0.5075
SXTY	3.667	2.559	3	3.420	0.591	0.566	0.590	0.583	1.008	−0.019	0.0391
SNDB	2.444	1.802	1	2.444	0.401	0.371	0.395	0.319	0.622	0.161	0.7652
GXFCG	1.778	1.262	0	1.652	0.166	0.161	0.168	0.213	0.268	−0.165	0.2405
GXGL	2.111	1.570	0	1.952	0.290	0.277	0.289	0.278	0.447	0.001	0.2013
GZLS	2.667	1.906	3	2.500	0.451	0.429	0.448	0.380	0.700	0.098	0.3489
SXYQ	2.111	1.244	0	1.872	0.168	0.159	0.165	0.111	0.295	0.107	0.0689
SNYL	2.667	1.820	1	2.507	0.404	0.384	0.401	0.315	0.653	0.143	0.0547
NXYC	2.444	1.633	0	2.333	0.333	0.321	0.333	0.325	0.559	0.018	0.0055^a^
NXQTX	3.778	2.321	0	3.422	0.494	0.474	0.495	0.519	0.892	−0.110	0.6880
NXZW	3.111	1.850	3	2.780	0.343	0.329	0.343	0.352	0.628	−0.072	0.9901
HBZJK	2.000	1.508	1	1.900	0.258	0.249	0.260	0.287	0.408	−0.135	0.9931
SNHZ	4.667	3.117	12	4.255	0.600	0.572	0.597	0.537	1.157	0.047	0.0189
HNJS	2.333	1.503	1	2.122	0.247	0.235	0.246	0.204	0.427	0.065	0.0602
FJLY	3.000	2.382	1	2.949	0.507	0.481	0.510	0.556	0.845	−0.146	0.0541
YNKM	2.889	1.713	3	2.592	0.383	0.365	0.381	0.352	0.636	0.033	0.0183
FJQZ	2.778	2.038	5	2.681	0.398	0.376	0.396	0.356	0.681	0.045	0.0774
ZJQZ	3.111	2.237	1	2.933	0.501	0.477	0.498	0.435	0.838	0.111	0.0011^a^
Mean	2.901	1.959	1.947	2.690	0.404	0.385	0.403	0.382	0.685	0.008	0.0000

Abbreviations: *A*_*r*_, allelic richness; *D*, gene diversity; *F*_IS_, inbreeding coefficient; *H*_*e*_, expected heterozygosity; *H*_*o*_, observed heterozygosity; HWE, Hardy–Weinberg Equilibrium; *I*, Shannon–Wiener index; *N*_*a*_, average number of alleles; *N*_*e*_, effective number of alleles; *P*_*a*_, private alleles richness; *uH*_*e*_, unbiased expected heterozygosity.

^a^ Indicates significant deviation from Hardy–Weinberg equilibrium after Bonferroni correction.

The genetic diversity results for the 19 geographical populations of *P. prunicola* are shown in Table 6. The average number of alleles (*N*_*a*_) was 1.778–4.667, with the highest value in SNHZ and the lowest value in GXFCG. The number of effective alleles (*N*_*e*_) ranged from 1.244 to 3.117. The smallest number of effective alleles was observed in SXYQ, followed by GXFCG, and the largest number was observed in SNHZ. The results were roughly the same as those for the average number of alleles.

After correcting for sample size differences in different geographical populations, the allelic richness of *P. prunicola* from different regions was 1.652–4.255 (mean value 2.690), with the highest value in SNHZ and the lowest value in GXFCG. The Shannon's index (*I*) ranged from 0.268 to 1.157, with the highest value in SNHZ and the lowest value in GXFCG.

As shown in Table 6, the genetic diversity of *P. prunicola* ranged from 0.166 to 0.620, with an average value of 0.404. Samples from Linfen of Shanxi Province (SXLF) had the highest genetic diversity (0.596), and samples from GXFCG had the lowest genetic diversity (0.166).

The observed heterozygosity (*H*_*o*_) ranged from 0.111 to 0.657, and the expected heterozygosity (*H*_*e*_) ranged from 0.159 to 0.596. The observed heterozygosity of some populations was lower than the expected heterozygosity, indicating that there was some degree of inbreeding. *F*_IS_ > 0 indicates that inbreeding occurred in the population, and the expected heterozygosity is greater than the observed heterozygosity. *F*_IS_ < 0 indicates that there is an excess of heterozygotes in the population and the expected heterozygosity is smaller than the observed heterozygosity. The results of the *F*_IS_ analysis were consistent with those for observed and expected heterozygosity analysis.

The *F*_ST_ and *N*_*m*_ matrices of 19 geographical populations of *P. prunicola* are shown in Table S1. The *F*_ST_ (0.098) between SXLF and Taiyuan of Shanxi Province (SXTY) populations is < 0.15. The degree of genetic differentiation is low, and there is a certain degree of gene flow. The *F*_ST_ (0.045) between Qingtongxia of Ningxia Province (NXQTX) and Zhongwei of Ningxia Province (NXZW) was < 0.05, indicating low genetic differentiation and substantial gene exchange between populations. The *F*_ST_ values for other pairs of populations were > 0.25, with obvious genetic differentiation and little gene exchange. The *F*_ST_ value for SXYQ and GXFCG was 0.704, showing that the populations were extremely differentiated with almost no genetic exchange. Overall, there was little gene flow among populations of *P. prunicola* and obvious genetic differentiation. The average genetic differentiation coefficient was 0.326, and the average gene flow was 0.690.

**FIGURE 1:**
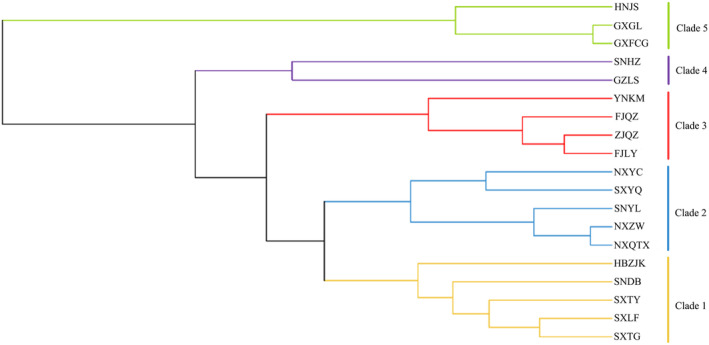
UPGMA phylogenetic tree of 19 populations of *P. prunicola*.

Microsatellite data for nine loci of *P. prunicola* were used to construct a UPGMA phylogenetic tree based on Nei's unbiased genetic distances between populations, as shown in Figure 1. The 19 geographical populations of *P. prunicola* can be divided into five clades. Shanxi (SXTG, SXTY and SXLF), Hebei Zhangjiakou (HBZJK) and Shaanxi Dingbian (SNDB) populations were genetically closely related, clustered into one branch (Clade 1). Ningxia (NXYC, NXZW and NXQTX) and SNYL and SXYQ populations clustered into one branch (Clade 2). YNKM and Fujian populations (FJLY and FJQZ) and the ZJQZ population formed a branch (Clade 4). GZLS and SNHZ formed one branch (Clade 4). HNJS and Guangxi populations (GXGL and GXFCG) were assigned to one branch (Clade 5). The clustering results for the 19 geographical populations were roughly consistent with the geographical division; the southern and northern populations were located in different clades.

**FIGURE 2:**
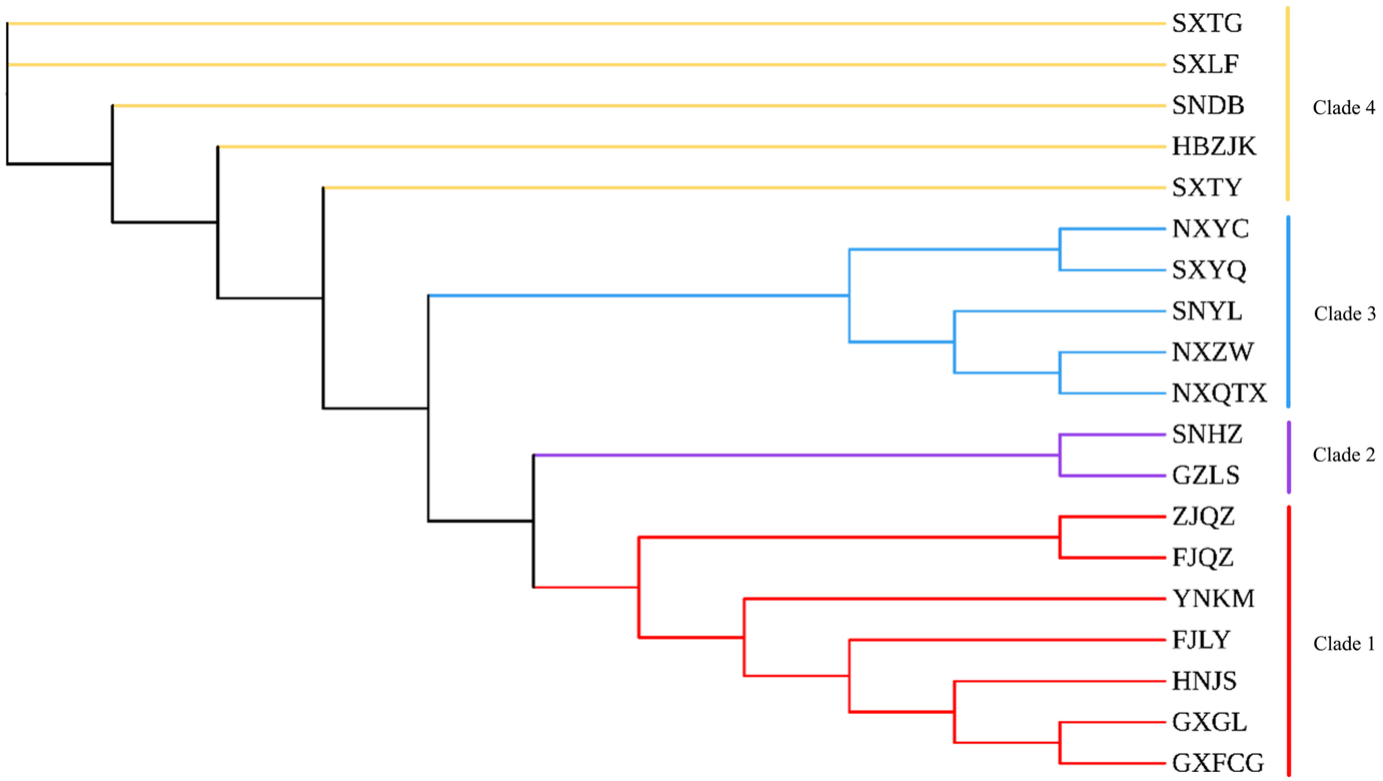
NJ phylogenetic tree of 19 populations of *P. prunicola*.

The NJ tree (Figure 2) constructed based on Nei's genetic distances revealed that the 19 geographical populations could be roughly assigned to four clusters. Populations in Zhejiang, Fujian, Yunnan, Hunan and Guangxi formed one cluster (Clade 1). GZLS and SNHZ populations were clustered (Clade 2). The Ningxia populations (NXYC, NXZW, NXQTX) and SNYL and SXYQ populations were clustered (Clade 3). The Shanxi populations (SXTG, SXTY, SXLF), HBZJK and SNDB formed a cluster (Clade 4). The clustering results were roughly the same as those in the UPMGA analysis. Guangxi and Hunan populations clustered into one lineage with southern populations, such as Fujian, Zhejiang and Yunnan. The overall clustering results showed an obvious north–south pattern.

**FIGURE 3:**
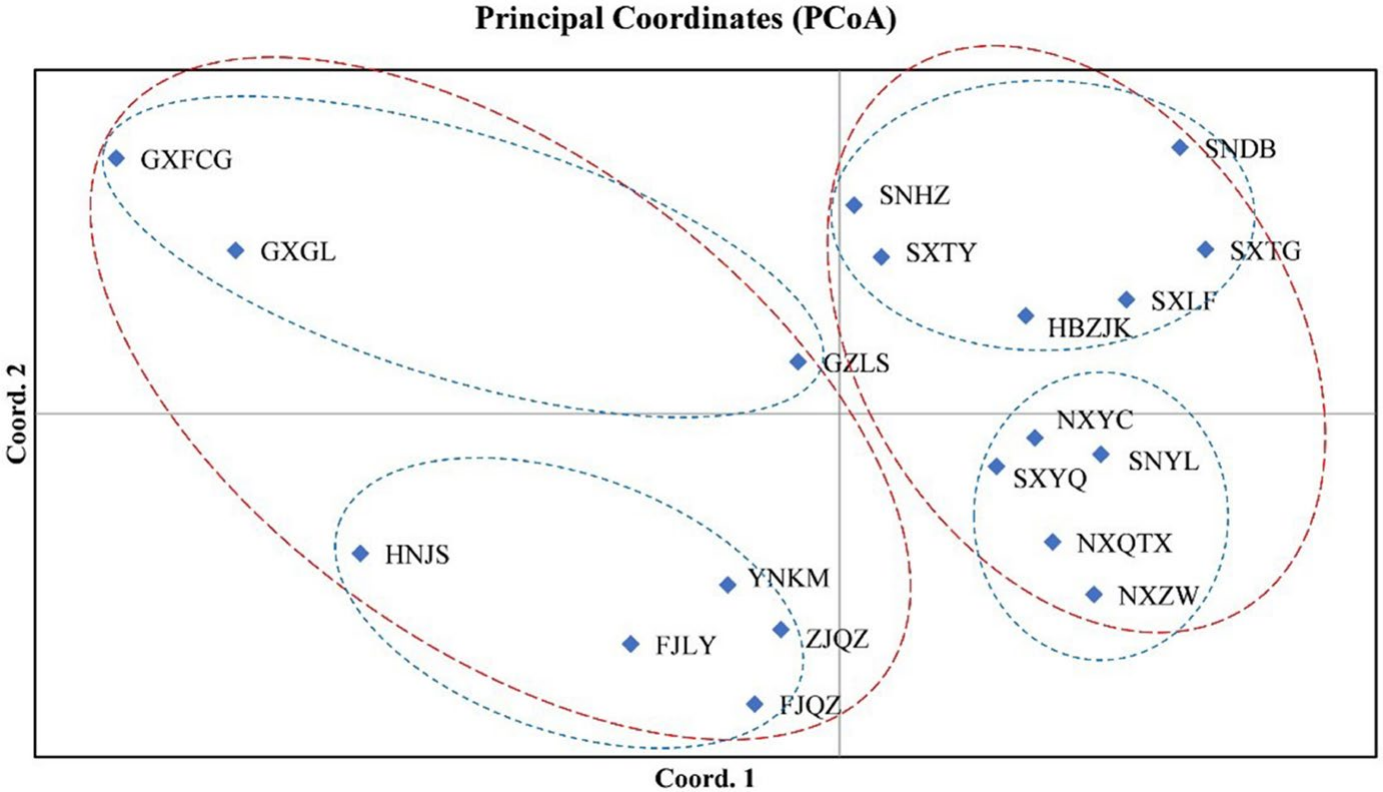
Principal coordinate analysis of *P. prunicola* populations.

The results of a PCoA of *P. prunicola* based on genetic and geographical distances are shown in Figure 3. Coord1, the first principal coordinate, explained 38.55% of the total variance; the 19 populations were divided into two major categories, one including GXFCG, GXGL, GZLS, HNJS, YNKM, FJLY, FJQZ, and ZJQZ (southern population) and another including SNHZ, SNYL, SNDB, SXTG, SXLF, SXYQ, SXTY, HBZJK, NXYC, NXQTX and NXZW (northern population).

Coord 2, the second principal coordinate, explained 16.77% of the total variance, and the cumulative contribution rate of Coord1 and Coord2 was 55.33%. The 19 populations could be divided into four major categories: (1) GXFCG, GXGL and GZLS, (2) HNJS, YNKM, FJLY, ZJQZ and FJQZ, (3) SXHZ, SXTY, HBZJK, SXLF, SXTG and SNDB and (4) NXYC, NXQTX, NXZW, SXYQ and SNYL. The overall clustering results were similar to the results of the phylogenetic analysis.

**FIGURE 4:**
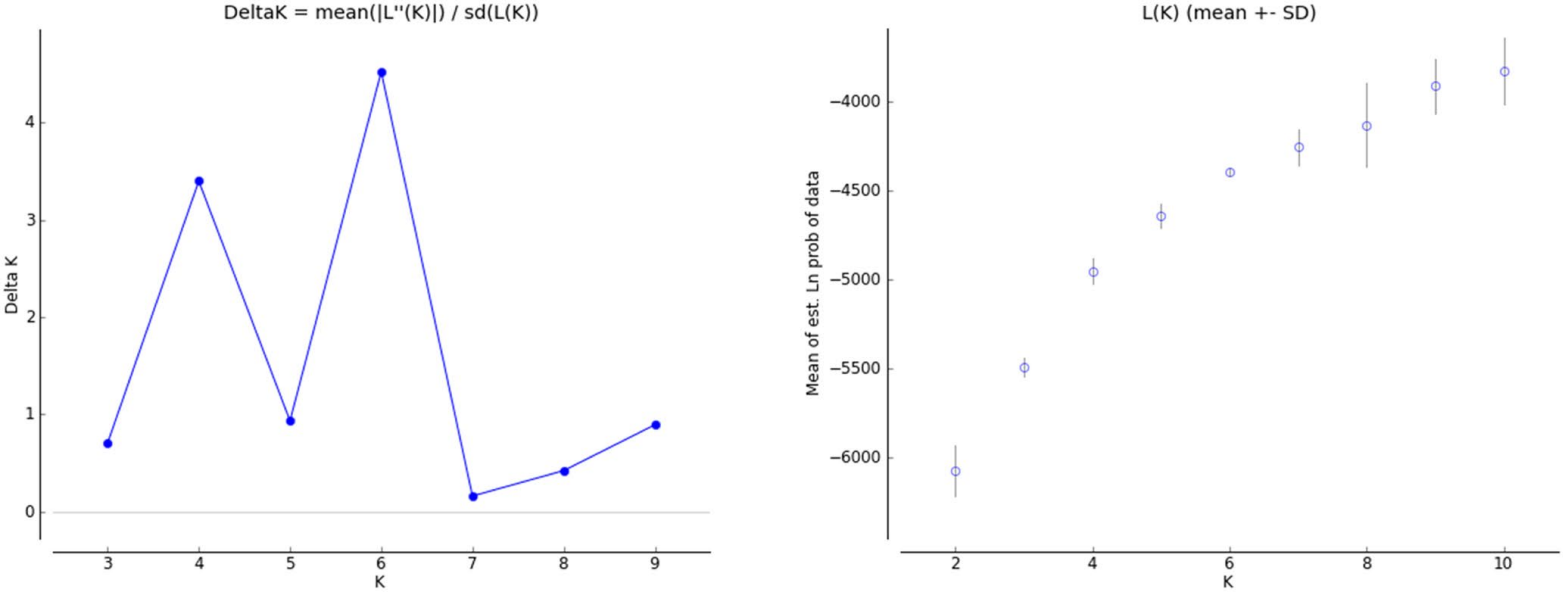
Relationship between ∆*K* and Ln *Pr*(*X|K*) values for different geographical populations of *P. prunicola*.

STRUCTURE software was used to analyse the genetic structure of different geographical populations of *P. prunicola*. Structure Harvester was used to calculate LnP (*K*), Δ*K* and the maximum possible value of *K*. The results are shown in Figure 4. The LnP (*K*) value increased as the *K* value increased, indicating that there was genetic differentiation among geographical populations of *P. prunicola*. Δ*K* had two peaks. When *K* = 6, Δ*K* was slightly larger than that when *K* = 4. However, according to the CLUMPP repeated sampling averaging process, the consistency of the 20 iteration results when *K* = 4 was 0.79235, compared with 0.65867 for *K* = 6.

**FIGURE 5:**
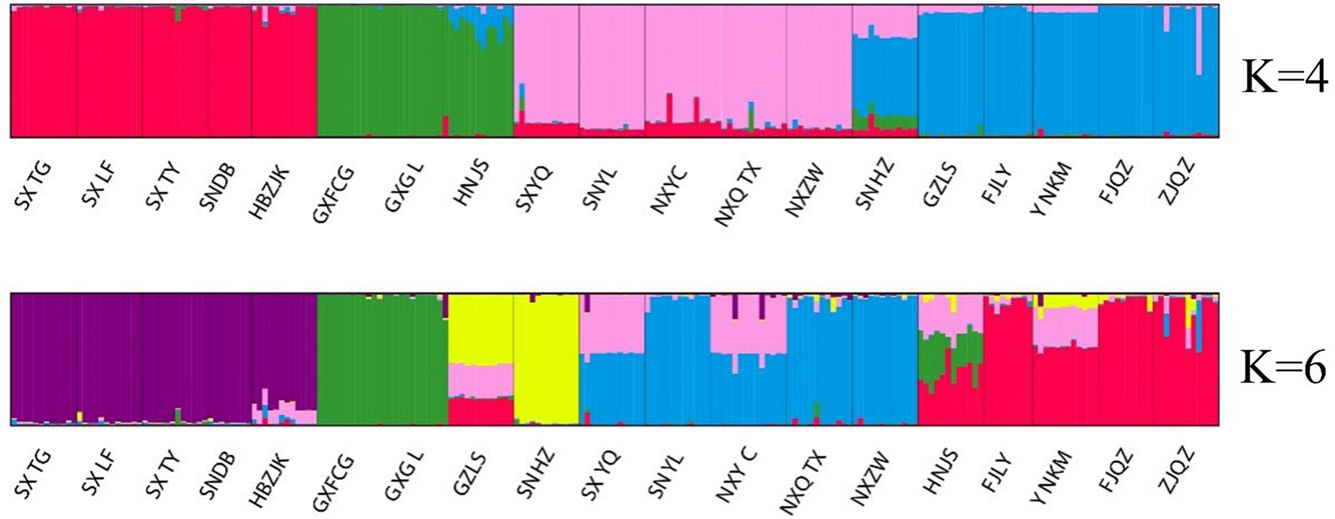
STRUCTURE analysis of 19 populations of *P. prunicola* at *K* = 4 and *K* = 6.

When *K* = 4 (Figure 5), *P. prunicola* was divided into the following four taxonomic clusters: (1) SXTG, SXLF, SXTY, SNDB and HBZJK (red), (2) GXFCG, GXGL and HNJS (green), (3) SXYQ, SNYL, NXYC, NXQTX and NXZW(pink) and (4) SNHZ, GZLS, FJLY, YNKM, FJQZ and ZJQZ (). The clustering results showed obvious north–south population differentiation, consistent with the PCoA results.

When *K* = 6 (Figure 5), the 19 geographical populations of *P. prunicola* were divided into five groups, indicating that this was not the optimal *K* value. The clustering results were roughly the same as those for *K* = 4, except the GZLS and SNHZ populations were assigned to a separate group. The clustering results were consistent with the phylogenetic analysis.

The structure analysis revealed that the 19 geographical populations of *P. prunicola* could be divided into four groups for AMOVA (Table 7). There was significant genetic differentiation between groups, between populations within groups and within populations of *P. prunicola* (*p* < 0.001). The fixation index values were all > 0.15. The differentiation coefficients between populations and within populations within groups were > 0.25, indicating extreme differentiation. The main source of genetic variation in *P. prunicola* was within populations.

**TABLE 7:** AMOVA of *P. prunicola* populations based on nine microsatellite loci.

Source of variation	*df*	Sum of squares	Variance components	Percentage of variation	Fixation indices
Among groups	3	356.373	0.85137 Va	23.09	*F*_CT_ = 0.23092 (*p* < 0.001)
Among populations within groups	15	385.432	1.03083 Vb	27.96	*F*_SC_ = 0.36354 (*p* < 0.001)
Within populations	423	763.377	1.80467 Vc	48.95	*F*_ST_ = 0.51051 (*p* < 0.001)
Total	441	1505.181	3.68688		

**FIGURE 6:**
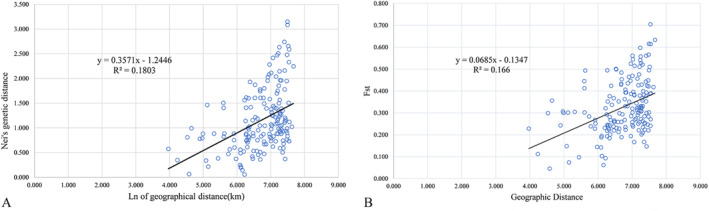
Mantel test of the relationship between geographical and genetic distances between *P. prunicola* populations based on SSR. (A) Mantel analysis of the natural logarithm of Nei's genetic distance versus geographical distance, (B) Mantel analysis of the natural logarithm of the genetic differentiation coefficient *F*_ST_ versus geographical distance.

GenAlEx was used to conduct Mantel correlation analyses based on Nei's genetic distance or *F*_ST_ (Y) and the natural logarithm (X) of geographical distances (*K*_*m*_) between populations of *P. prunicola*. The following regression equations were obtained Y = 0.3571x−1.2446 and Y = 0.0685x−0.1347, respectively (Figure 6). The results show that there was a significant positive correlation between the genetic distance and geographical distance between populations of *P. prunicola* in China (*R* ^2^ = 0.1803, *p* < 0.05). At the same time, there was a significant linear relationship between the fixation coefficient *F*_ST_ and geographical distance (*R* ^2^ = 0.166, *p* > 0.05). A lack of gene flow caused by geographical isolation shaped the existing genetic architecture.

Based on three models, IAM, TPM and SMM, bottleneck detection was performed using the model‐shift and Wilcoxon signed rank test implemented in Bottleneck software. When the model‐shift test result shows an L‐shaped distribution, it usually indicates that a bottleneck has not occurred and the allele distribution frequency of microsatellite loci does not deviate from the mutation‐migration balance. The model‐shift test results are shown in Table 8. Except for the SNDB, NXYC, FJLY, FJQZ and ZJQZ populations, the other populations showed L‐shaped distributions. However, using the Wilcoxon signed‐rank test, none of the above populations showed a significant excess in heterozygosity. The populations SXTG, SXYQ and NXZW had a significant excess in heterozygosity; however, the model‐shift test results showed L‐shaped distributions. Taken together, *P. prunicola* populations may not have experienced recent genetic bottleneck events in China.

**TABLE 8:** Bottleneck test of *P. prunicola*.

Code	IAM	TPM	SMM	Model‐shift
SXTG	0.91016	0.03711*	0.02734*	L
SXLF	0.00391**	0.42578	0.57031	L
SXTY	0.00977**	0.49609	0.57031	L
SNDB	0.46094	0.84375	0.84375	Shifted
GXFCG	0.62500	0.21875	0.21875	L
GXGL	0.06250	0.62500	0.81250	L
GZLS	0.05469	0.54688	0.84375	L
SXYQ	0.03906*	0.01563*	0.01563*	L
SNYL	0.38281	1.00000	1.00000	L
NXYC	0.38281	0.64063	0.38281	Shifted
NXQTX	0.82031	0.49609	0.49609	L
NXZW	0.35938	0.00977*	0.00977**	L
HBZJK	0.68750	0.81250	0.81250	L
SNHZ	0.38281	0.64063	0.46094	L
HNJS	0.56250	0.15625	0.07813	L
FJLY	0.03906*	0.46094	0.54688	Shifted
YNKM	0.84375	0.25000	0.25000	L
FJQZ	0.29688	0.81250	0.81250	Shifted
ZJQZ	0.03906*	0.54688	0.74219	Shifted

* Indicates two‐tailed test significance level *p* < 0.05.

** Indicates highly significant *p* < 0.01.

### 3.3 Genetic Diversity Analysis of Different Geographic Populations in COI Gene Fragments

In this study, COI gene fragments were successfully amplified from 329 samples of 19 geographical populations of *P. prunicola* collected in China (GenBank accession number: PP995286–PP995614); additionally, two COI sequences of *P. prunicola* from Korea (Park et al. 2011, GenBank accession: HM474347 and HM474346) were obtained from GenBank. After splicing, proofreading and alignment, 626 bp were obtained for subsequent analyses. There were no insertions or deletions in any sequences, and there were 65 polymorphic sites, accounting for 12.5% of the total sites, including 3 sites with a single mutation nucleotide polymorphism and 62 parsimony‐informative sites. The (A + T) % of all sequences was 82.428%–84.026%, showing a very obvious A/T bias.

In China, the overall genetic diversity in *P. prunicola* was low. The overall haplotype diversity (*H*_*d*_) was 0.905 ± 0.006, nucleotide diversity (*π*) was 0.0175 ± 0.00096, and average number of nucleotide differences (*K*) was 10.957 (Table 9). The genetic variation within populations differed. The SXTG population had four haplotypes (*H*_*d*_ was 0.542 ± 0.123 and *π* was 0.00163 ± 0.00047), followed by GXGL and NXZW, which had three haplotypes (*H*_*d*_ values were 0.279 ± 0.123 and 0.689 ± 0.043, *π* values were 0.00046 ± 0.00021 and 0.00297 ± 0.0002 respectively). FJQZ, SXTY and ZJQZ contained two haplotypes (*H*_*d*_ values were 0.2 ± 0.154, 0.1 ± 0.088 and 0.233 ± 0.123, *π* values were 0.00032 ± 0.00025, 0.00032 ± 0.00028 and 0.00447 ± 0.00241, respectively). The remaining populations had only one haplotype, and *H*_*d*_, *π* and *K* values were 0.

**TABLE 9:** Genetic diversity of *P. prunicola* populations based on mitochondrial COI haplotypes.

Population	*H*	Haplotype distribution	*H*_*d*_	*π*	*K*	*S*
South Korea	1	Hap_1(2)	0	0	0	0
FJLY	1	Hap_2(9)	0	0	0	0
FJQZ	2	Hap_3(9), Hap_4(1)	0.2 ± 0.154	0.00032 ± 0.00025	0.2	1
GXFCG	1	Hap_5(20)	0	0	0	0
GXGL	3	Hap_5(17), Hap_6(1), Hap_7(2)	0.279 ± 0.123	0.00046 ± 0.00021	0.289	2
GZLS	1	Hap_2(20)	0	0	0	0
HBZJK	1	Hap_8(16)	0	0	0	0
HNJS	1	Hap_5(20)	0	0	0	0
NXQTX	1	Hap_9(20)	0	0	0	0
NXYC	1	Hap_10(20)	0	0	0	0
NXZW	3	Hap_11(5), Hap_12(7), Hap_13(8)	0.689 ± 0.043	0.00297 ± 0.00020	1.858	4
SNDB	1	Hap_14(8)	0	0	0	0
SNHZ	1	Hap_15(20)	0	0	0	0
SNYL	1	Hap_12(20)	0	0	0	0
SXLF	1	Hap_16(19)	0	0	0	0
SXTG	4	Hap_16(12), Hap_17(1), Hap_18(3), Hap_19(2)	0.542 ± 0.123	0.00163 ± 0.00047	1.02	5
SXTY	2	Hap_16(19), Hap_20(1)	0.100 ± 0.088	0.00032 ± 0.00028	0.2	2
SXYQ	1	Hap_21(20)	0	0	0	0
YNKM	1	Hap_22(13)	0	0	0	0
ZJQZ	2	Hap_2(14), Hap_21(2)	0.233 ± 0.126	0.00447 + −0.00241	2.8	12
All	22		0.905 ± 0.006	0.01750 + −0.00096	10.957	65

*Note: H* is the number of haplotypes, *H*_*d*_ is haplotype diversity, *π* is nucleotide diversity, *K* is the average number of nucleotide differences, and *S* is the number of polymorphic sites.

**FIGURE 7:**
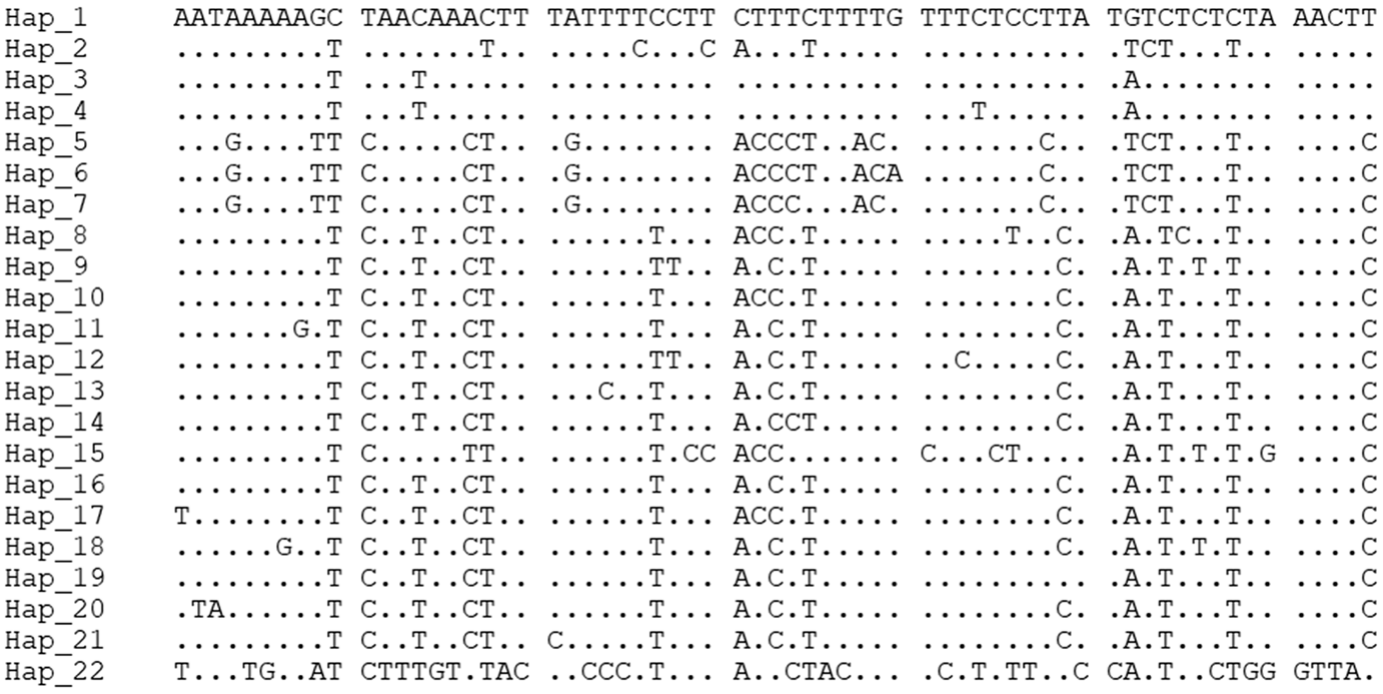
Variable sites and haplotypes of COI in *P. prunicola*.

In total, 331 samples from 20 geographical populations of *P. prunicola* were collected, and 22 haplotypes were detected in the mitochondrial COI gene fragment using DnaSP 6.12.03 (Figure 7). Among these, 17 haplotypes were unique haplotypes, and 5 haplotypes were unique. Haplotypes (Hap_2, Hap_5, Hap_12, Hap_16 and Hap_21) were shared, among which Hap_2 was the shared among FJLY, GZLS and ZJQZ (accounting for 13.0% of the total). Hap_5 was detected in GXFCG, GXGL and GZLS (accounting for 17.2%). Haplotype Hap_12 was shared by NXZW and SNYL, with a number of 27 (8.2%). Hap_16 was shared by populations SXLF, SXTG and SXTY (15.1%). Hap_21 was shared by populations SXYQ and ZJQZ (accounting for 6.6% of the total number of samples).

**FIGURE 8:**
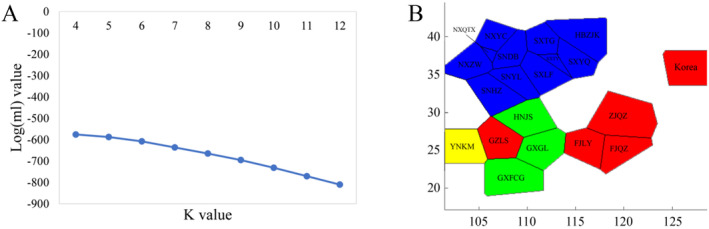
Analysis of spatial clustering using BAPS.

BAPS (Bayesian Analysis of Population Structure) is a Bayesian analysis method based on the principle of spatial genetic mixing. It uses a spatial clustering model to combine the spatial location of population samples with haplotype data to estimate the change in the log marginal likelihood value (mL) when the number of groups *K* is different. The *K* value corresponding to the maximum mL is used as the optimal value, as shown in Figure 8A.

Based on the mitochondrial haplotype data for *P. prunicola*, when *K* = 4, the maximum log (mL) was −575.442, indicating that the 20 populations could be divided into 4 groups. Cluster 1 included Korea, FJLY, FJQZ, GZLS and ZJQZ (red); Cluster 2 included GXFCG, GXGL and HNJS (green); Cluster 3 included HBZJK, NXQTX, NXYC, NXZW, SNDB, SNHZ, SNYL, SXLF, SXTG, SXTY and SXYQ (blue); Cluster 4 included YNKM (yellow) (Figure 8B, the abscissa and ordinate represent the longitude and latitude, respectively).

**FIGURE 9:**
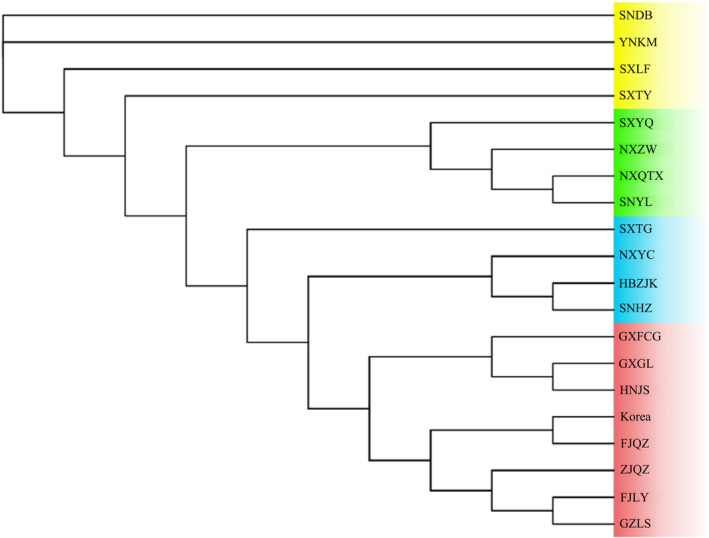
Neighbour‐Joining tree of *P. prunicola* populations based on haplotype genetic distances.

A NJ phylogenetic tree based on the genetic distances between haplotypes of 20 geographical populations of *P. prunicola*, as shown in Figure 9. The populations were divided into four taxonomic clusters: one (yellow) including SXDB, YNKM, SXLF and SXTY, the second cluster (green) including SXYQ, NXZW, NXQTX and SNYL, the third cluster (blue) including SXTG, NXYC, HBZJK and SNHZ, and the final cluster (pink) including GXFCG, GXGL, HNJS, Korea, FJQZ, ZJQZ, FJLY and GZLS. The NJ phylogenetic tree based on genetic distances between haplotypes clearly showed that except for the YNKM population, all southern populations clustered into a single large branch, while the remaining northern populations were divided into different lineages. The results were similar to those of the BAPS analysis, in which the Yunnan populations formed a separate branch.

**FIGURE 10:**
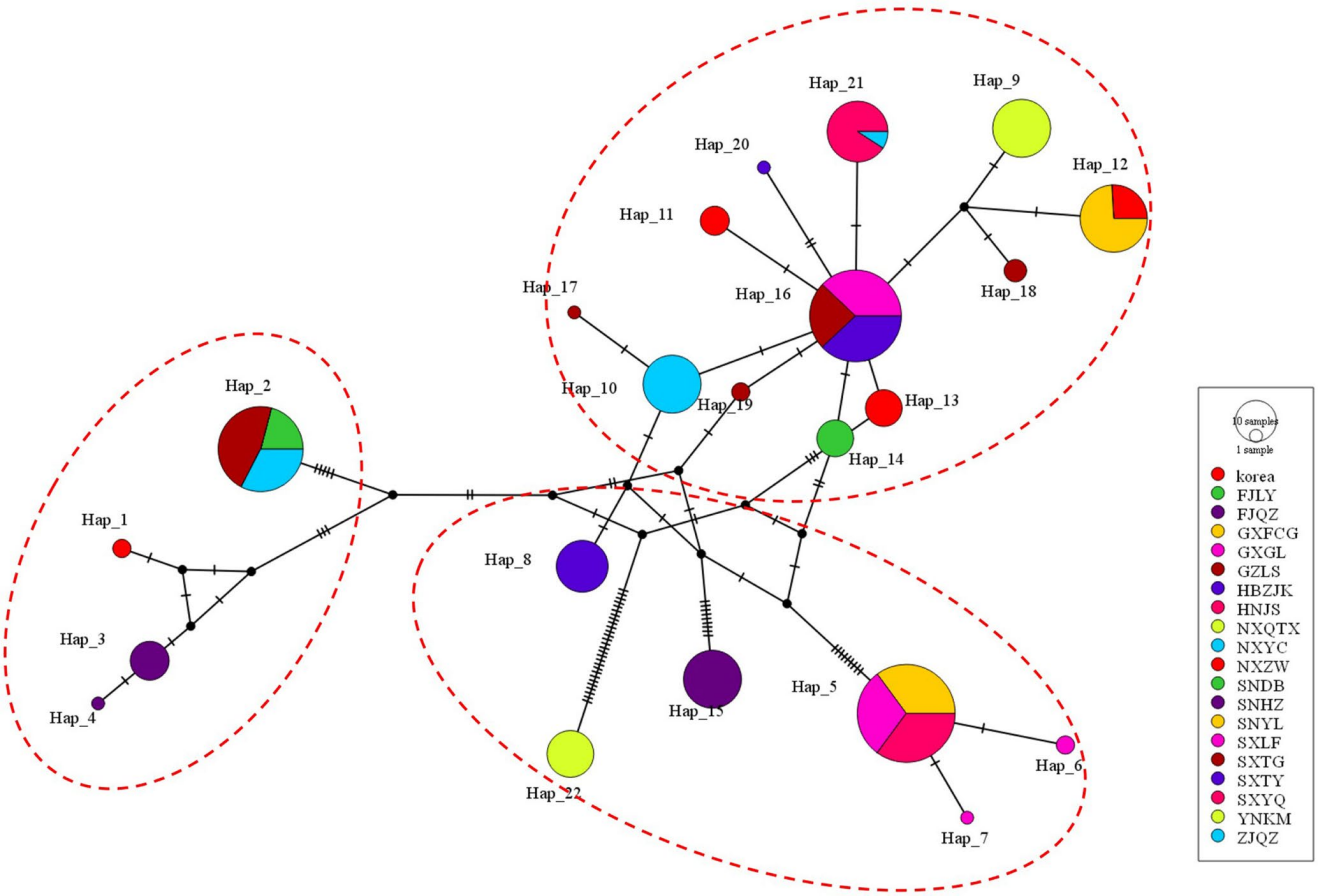
Haplotype network of different populations of *P. prunicola*.

The results of a NJ network diagram analysis of haplotypes in different populations of *P. prunicola* evaluated using PopART are shown in Figure 10. The short horizontal lines represent the mutations. The *P. prunicola* population could be roughly divided into three taxonomic clusters. Cluster 1 mainly included haplotypes Hap_8–13 and Hap_14–20, which are mainly northern populations. Cluster 2 mainly included Hap_1–4, which are Korean populations and the Fujian population. Cluster 3 mainly included Hap_5–7, Hap_15 and Hap_22, mainly the Guangxi, Shaanxi Hanzhong and Yunnan populations. Based on the large number of base mutations between Hap_22 and other haplotypes, Hap_22 could be divided into a distinct category. The clustering results were roughly consistent with the BAPS spatial grouping results.

**FIGURE 11:**
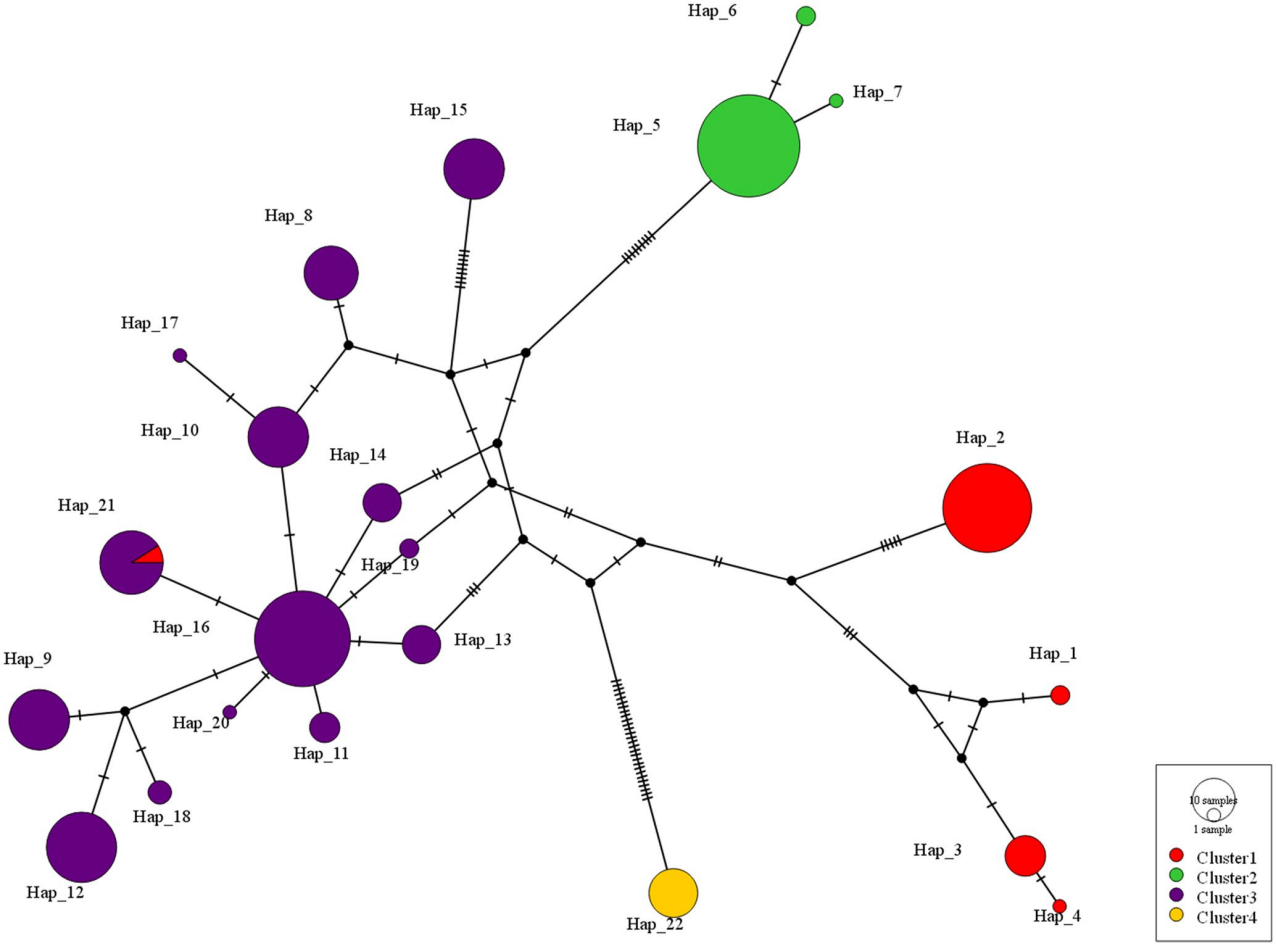
Network of haplotypes in different groups of *P. prunicola*. Each circle in the figure represents a haplotype. The size of the circle represents the number of samples of the haplotype. Different colours represent different populations.

Based on the BAPS spatial grouping results, an adjacency network diagram was constructed for the haplotypes of different geographical populations of *P. prunicola*. As shown in Figure 11, the distribution of each haplotype was relatively scattered, and the genetic variation between haplotypes was relatively high. There was obvious differentiation between groups and a relatively fixed genetic structure, forming an obvious geographical structure. Haplotypes in the purple group showed a weak star‐like distribution, centred on haplotype Hap_16, indicating that the population with Hap_16 may have experienced an expansion and is an ancestral haplotype. In the green group, Hap_5 was the central node shared among GXFCG, GXGL and HNJS, indicating that the three populations have a similar genetic structure and may be an ancestral group.

Using Arlequin 3.5.2.2, haplotype data were grouped according to the geographical origin. Pairwise differences were used to calculate the genetic differentiation coefficient (*F*_ST_) and gene flow (*N*_*m*_ = (1/*F*_ST_−1)/4) between populations.

According to *N*_*m*_ = (1/*F*_ST_−1)/4, we can derive the gene flow between groups. A higher degree of genetic differentiation between populations indicates less gene exchange. *F*_ST_ can be divided into different levels as follows: *F*_ST_ ≤ 0.05 (low degree of differentiation), 0.05 < *F*_ST_ ≤ 0.25 (medium degree of differentiation), and *F*_ST_ > 0.25 (extreme differentiation). Based on this analysis (Table 10), the degree of genetic differentiation between each group was extremely high, with almost no gene exchange.

**TABLE 10:** Genetic differentiation (lower diagonal) and gene flow (upper diagonal) among groups of *P. prunicola*.

	Cluster1	Cluster2	Cluster3	Cluster4
Cluster1		0.039148	0.117118	0.025203
Cluster2	0.86461		0.061364	0.000501
Cluster3	0.68098	0.80292		0.028328
Cluster4	0.90842	0.99800	0.89822	

According to the BAPS grouping results, 20 geographical populations of *P. prunicola* were divided into four groups for AMOVA (Table 11). The proportions of genetic variation among groups, between populations within groups and within populations of *P. prunicola* were 77.65%, 20.32% and 2.03% of the total variation, respectively, indicating that the genetic variation was mainly between groups. The values of *F*_CT_ (between groups), *F*_SC_ (between populations within a group) and *F*_ST_ (within a middle group) were 0.7765, 0.90911 and 0.97969 respectively, indicating that *P. prunicola* exhibits extreme differentiation among groups, between populations within groups and within populations.

**TABLE 11:** AMOVA of 20 populations of *P. prunicola*.

Source of variation	*df*	Sum of squares	Variance components	Percentage of variance	Fixation indices
Among groups	3	1302.904	6.49785	77.65	*F*_CT_ = 0.77650 *p* < 0.001
Among populations within groups	16	452.139	1.70031	20.32	*F*_SC_ = 0.90911 *p* < 0.001
Within populations	311	52.867	0.16999	2.03	*F*_ST_ = 0.97969 *p* < 0.001
Total	330	1807.909	8.36815		

**FIGURE 12:**
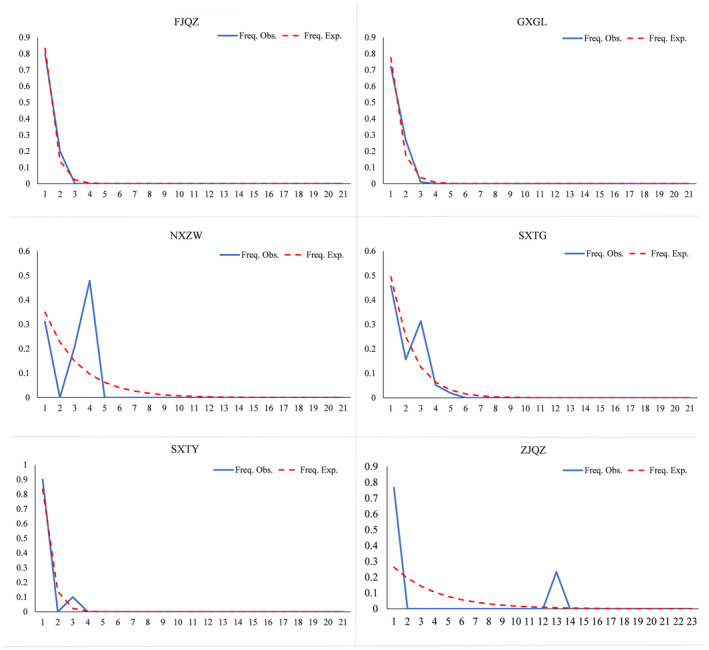
Nucleotide mismatch distribution of *P. prunicola*.

A nucleotide mismatch analysis was conducted. Since most populations had only one haplotype, the nucleotide mismatch analysis was only performed for populations with two or more haplotypes. The results are shown in Figure 12, revealing that the observed frequency distributions of the FJQZ and GXGL populations were unimodal curves, while those for the NXZW, SXTG, SXTY and ZJQZ populations were multimodal curves. Therefore, the FJQZ and GXGL populations have experienced rapid expansion, and the last four populations remained relatively stable. The sum of squared deviations (*SSD*) and roughness index (*R*) results are shown in Table 12. Neither the FJQZ nor GXGL populations reached a significant level, indicating a lack of deviations from the assumptions of the population expansion model. A population may have experienced population expansion in the past.

**TABLE 12:** Neutrality test of *P. prunicola* populations.

Code	Tajima's *D*	Fu's *Fs*	SSD	*R*
FJQZ	−1.11173	−0.33931	0.00029	0.40000
GXGL	−1.14070	−1.20637*	0.00172	0.27150
NXZW	1.87542	2.96118	0.10920*	0.44219
SXTG	−0.94515	−0.08067	0.02400	0.18480
SXTY	−1.51284*	−0.02545	0.00550	0.83000
ZJQZ	−0.86235	6.36564	0.04423	0.69667
All	−0.18487	0.38375	0.029470	0.02007

* *p* < 0.05.

The results of neutrality tests for the six geographical populations of *P. prunicola* based on mitochondrial COI are shown in Table 12.

The Fu's *Fs* and Tajima's *D* values of the GXGL and SXTY populations were significantly negative; however, the *SSD* and *R* values did not exceed 0.05, indicating a lack of population expansion. Except for NXZW, all Tajima's *D* values were negative; however, only the value for the SXTY population was significant (*p* < 0.05). In *Fu's Fs* test, GXGL, SXTG and SXTY had negative *Fs* values; however, only the value for GXGL was significant (*p* < 0.05). Some studies have shown that mismatch distribution analyses easily lose historical information in large data sets (Felsenstein 1992), while Tajima's *D* and Fu's *Fs* neutrality tests, especially the latter test, are useful in detecting signals of population growth. In particular, if Fu's *Fs* is significant but Tajima's *D* is not significant, this indicates population expansion (Ramos‐Onsins and Rozas 2002). Moreover, Tajima's *D* test usually reflects ancient population expansion events, and Fu's *Fs* test method usually reflects recent population expansion events. Therefore, combined with the results of the mismatch analysis, the GXGL population of *P. prunicola* scales may have experienced population expansion in the recent past, while the SXTY population may have experienced an ancient population expansion, while the remaining populations have remained relatively stable.

## 4 Discussion

This study employed microsatellite and mitochondrial COI markers to investigate the genetic diversity and structure of *P. prunicola* populations across China. The results reveal key patterns of genetic differentiation and provide insights into the population dynamics and evolutionary history of this species. The analysis showed that the genetic diversity of *P. prunicola* populations was generally low. Key metrics such as the effective number of alleles (*N*_*e*_: 1.244–3.117), allele richness (*A*_*r*_: 1.652–4.255), genetic diversity (*D*: 0.166–0.620) and heterozygosity levels (*H*_*e*_: 0.159–0.596, *H*_*o*_: 0.213–0.657) indicated variations among populations. Populations from SNHZ and SXLF displayed higher genetic diversity, which could be attributed to more stable environmental conditions or historical gene flow, while GXFCG exhibited the lowest diversity, likely reflecting geographic isolation or demographic bottlenecks (Frankham et al. 2002). Microsatellite marker analyses confirmed high polymorphism (PIC: 0.560–0.896) with no significant linkage disequilibrium (LD) or deviations from Hardy–Weinberg equilibrium (HWE), suggesting effective marker selection (Botstein et al. 1980). The positive inbreeding coefficients (FIS) further indicate a degree of inbreeding, leading to heterozygosity loss and limited gene flow in certain populations.

Measures of genetic differentiation, such as FST values exceeding 2.5 between most populations, pointed to high levels of genetic structure and limited gene exchange. Gene flow (*N*_*m*_ < 1) was similarly constrained, with only nearby populations in the same province showing evidence of genetic exchange. This isolation could be attributed to the limited dispersal ability of *P. prunicola* and the geographical barriers such as the Qinling Mountains and Huaihe River, which were identified as key dividing lines in population structure (Slatkin 1985; Mantel 1967). Phylogenetic analyses, principal coordinates analysis (PCoA), and structure analyses revealed distinct north–south population differentiation, with further subdivision into regional clusters. Northern populations were grouped into two branches, while southern populations exhibited more genetic variation. Mantel tests demonstrated a significant positive correlation between genetic and geographic distances, reinforcing the role of geographical isolation in shaping population structure (Wright 1943). A population bottleneck analysis and the Wilcoxon signed‐rank test showed that *P. prunicola* has not experienced a genetic bottleneck event in the short term in China, and the population genetic structure is relatively stable. These results also provide evidence that *P. prunicola* is native to the temperate zone of China or Japan (Miller and Davidson 2005).

Haplotype analyses identified 22 haplotypes, with low overall diversity (*H*_*d*_: 0.905, *π*: 0.0175). The low haplotype and nucleotide diversity in most populations suggests historical bottlenecks or founder events, limiting genetic variation (Nei, Maruyama, and Chakraborty 1975). Some populations, such as GXGL, exhibited recent expansions, while others remained stable over time. The lack of haplotype diversity in certain populations could reflect limited sampling or a recent reduction in genetic variability (Excoffier, Laval, and Schneider 2005). In an analysis of mitochondrial haplotypes of *P. prunicola*, a total of 22 haplotypes were detected, of which 5 haplotypes were shared, indicating that these populations may share the same origin and have similar genetic structures. Based on haplotype data, BAPS, haplotype network evolution diagrams and phylogenetic trees were used to analyse the genetic structure of *P. prunicola*. The northern population of *P. prunicola* may have expanded from the SXTY, SXLF and SXTG populations. The Korean population may have the same genetic background as populations in the southeastern coastal areas of China.

The findings highlight the vulnerability of *P. prunicola* populations to environmental and anthropogenic pressures due to their low genetic diversity and limited dispersal ability. This isolation increases the risk of local extinction and reduces adaptability to changing environments (Frankham, Bradshaw, and Brook 2014). In a relatively stable habitat, there is little genetic exchange between populations, making populations highly susceptible to environmental and human factors as well as the influence of genetic background, resulting in different genetic structures (Fenton et al. 2010; Torriani et al. 2010). Future studies should incorporate broader sampling across geographic ranges and employ additional molecular markers to explore deeper phylogeographic patterns. The role of host‐plant specificity and environmental variables in shaping genetic structure also warrants further investigation.

## 5 Conclusion

The genetic diversity of white prunicola scale in China was low. Gene flow among populations was low. A clear geographic genetic structure was detected. The 19 populations of the white prunicola scale in China could be divided into four groups and showed an obvious north–south distribution pattern. There was substantial genetic differentiation between groups, with little gene exchange. Analyses of diversity based on COI markers yielded similar results to those of microsatellite analyses. The distribution range of the white prunicola scale in China is very wide, and the sampling range in this study is relatively limited, lacking samples from East China, Central China, Northeast China and other regions. Therefore, in future research, it is necessary to increase the sampling range and amount of the white prunicola scale, further improve the research methods of genetic diversity and genetic structure of its different populations, and improve the reliability of research results. This study revealed the genetic diversity and structure of the white prunicola scale, but there is a lack of research on the effects of different environmental factors on its distribution range, population structure and population dynamics. Therefore, in the future, research in these areas should be increased to understand the differentiation pathways and evolutionary mechanisms of the white prunicola scale in China, providing a more comprehensive and richer theoretical basis for the comprehensive management of this insect.

## Author Contributions

**Minmin Niu:** conceptualization (lead), formal analysis (lead), funding acquisition (equal), methodology (lead), writing – original draft (lead), writing – review and editing (equal). **Dengen Fu:** data curation (equal), formal analysis (equal). **Haoyang Wang:** data curation (equal), formal analysis (equal). **Yun Liu:** investigation (supporting), software (equal). **Xuanxing Du:** conceptualization (supporting), software (equal). **Qing Zhao:** project administration (lead), supervision (lead). **Jiufeng Wei:** funding acquisition (lead), supervision (supporting), writing – review and editing (supporting).

## Conflicts of Interest

The authors declare no conflicts of interest.

## Supporting information


Table S1

## Data Availability

COI data of 329 samples of *P. prunicola* collected in China are deposited to NCBI Nucleotide Database (GenBank accession number: PP995286–PP995614).
