# Supplementary material for: Microsatellite and Mitochondrial COI Provide Novel Insights Into the Population Genetic Structure of White Prunicola Scale (Pseudaulacaspis prunicola) in China
Source: Ecol Evol. 2025 Jan 20;15(1):e70865. doi: 10.1002/ece3.70865 (PMC11745613; doi:10.1002/ece3.70865)
Supplement: Supplementary file 1 — Table S1 [file ECE3-15-e70865-s001.docx]

S1 Table Matrices of genetic differentiation (*F*_ST_) (lower left) and gene flow (upper right) among populations of *Pseudaulacaspis prunicola*

| 种群代码  Population  Code | SXTG | SXLF | SXTY | SNDB | GXFCG | GXGL | GZLS | SXYQ | SNYL | NXYC | NXQTX | NXZW | HBZJK | SNHZ | HNJS | FJLY | YNKM | FJQZ | ZJQZ |
| --- | --- | --- | --- | --- | --- | --- | --- | --- | --- | --- | --- | --- | --- | --- | --- | --- | --- | --- | --- |
| SXTG |  | 3.138 | 1.985 | 1.030 | 0.255 | 0.336 | 0.611 | 0.452 | 0.768 | 0.638 | 0.991 | 0.693 | 0.745 | 0.830 | 0.310 | 0.566 | 0.584 | 0.506 | 0.721 |
| SXLF | 0.074 |  | 2.313 | 1.480 | 0.315 | 0.401 | 0.801 | 0.578 | 0.847 | 0.799 | 1.045 | 0.685 | 0.770 | 0.894 | 0.439 | 0.883 | 0.817 | 0.671 | 1.039 |
| SXTY | 0.112 | 0.098 |  | 1.077 | 0.395 | 0.512 | 0.720 | 0.586 | 0.781 | 0.840 | 1.105 | 0.716 | 0.798 | 0.927 | 0.578 | 0.947 | 0.762 | 0.741 | 1.247 |
| SNDB | 0.195 | 0.145 | 0.188 |  | 0.192 | 0.239 | 0.478 | 0.460 | 0.684 | 0.583 | 0.779 | 0.570 | 0.719 | 0.528 | 0.226 | 0.414 | 0.450 | 0.307 | 0.504 |
| GXFCG | 0.495 | 0.443 | 0.387 | 0.566 |  | 2.428 | 0.256 | 0.105 | 0.198 | 0.157 | 0.240 | 0.156 | 0.145 | 0.298 | 0.587 | 0.324 | 0.249 | 0.196 | 0.275 |
| GXGL | 0.426 | 0.384 | 0.328 | 0.511 | 0.093 |  | 0.356 | 0.169 | 0.288 | 0.230 | 0.333 | 0.223 | 0.196 | 0.386 | 0.630 | 0.431 | 0.301 | 0.266 | 0.365 |
| GZLS | 0.290 | 0.238 | 0.258 | 0.344 | 0.494 | 0.413 |  | 0.410 | 0.684 | 0.555 | 0.748 | 0.526 | 0.460 | 0.844 | 0.312 | 0.872 | 0.415 | 0.627 | 0.773 |
| SXYQ | 0.356 | 0.302 | 0.299 | 0.352 | 0.704 | 0.597 | 0.379 |  | 0.800 | 0.890 | 0.713 | 0.459 | 0.257 | 0.327 | 0.209 | 0.432 | 0.344 | 0.376 | 0.532 |
| SNYL | 0.246 | 0.228 | 0.242 | 0.268 | 0.558 | 0.464 | 0.268 | 0.238 |  | 0.830 | 2.188 | 1.508 | 0.507 | 0.579 | 0.251 | 0.523 | 0.408 | 0.520 | 0.681 |
| NXYC | 0.282 | 0.238 | 0.229 | 0.300 | 0.614 | 0.521 | 0.310 | 0.219 | 0.231 |  | 0.843 | 0.565 | 0.333 | 0.491 | 0.236 | 0.576 | 0.403 | 0.392 | 0.630 |
| NXQTX | 0.201 | 0.193 | 0.184 | 0.243 | 0.510 | 0.429 | 0.250 | 0.260 | 0.103 | 0.229 |  | 5.286 | 0.601 | 0.866 | 0.382 | 0.886 | 0.705 | 0.786 | 1.245 |
| NXZW | 0.265 | 0.267 | 0.259 | 0.305 | 0.616 | 0.528 | 0.322 | 0.352 | 0.142 | 0.307 | 0.045 |  | 0.386 | 0.603 | 0.283 | 0.634 | 0.523 | 0.674 | 0.869 |
| HBZJK | 0.251 | 0.245 | 0.238 | 0.258 | 0.633 | 0.561 | 0.352 | 0.493 | 0.330 | 0.429 | 0.294 | 0.393 |  | 0.397 | 0.212 | 0.386 | 0.348 | 0.340 | 0.549 |
| SNHZ | 0.231 | 0.218 | 0.212 | 0.321 | 0.456 | 0.393 | 0.229 | 0.433 | 0.302 | 0.337 | 0.224 | 0.293 | 0.386 |  | 0.340 | 0.581 | 0.462 | 0.557 | 0.665 |
| HNJS | 0.446 | 0.363 | 0.302 | 0.525 | 0.299 | 0.284 | 0.445 | 0.544 | 0.499 | 0.515 | 0.396 | 0.469 | 0.541 | 0.424 |  | 1.072 | 0.716 | 0.507 | 0.814 |
| FJLY | 0.307 | 0.221 | 0.209 | 0.377 | 0.435 | 0.367 | 0.223 | 0.367 | 0.324 | 0.302 | 0.220 | 0.283 | 0.393 | 0.301 | 0.189 |  | 0.992 | 1.438 | 3.800 |
| YNKM | 0.300 | 0.234 | 0.247 | 0.357 | 0.501 | 0.454 | 0.376 | 0.421 | 0.380 | 0.383 | 0.262 | 0.324 | 0.418 | 0.351 | 0.259 | 0.201 |  | 0.738 | 1.451 |
| FJQZ | 0.331 | 0.271 | 0.252 | 0.449 | 0.561 | 0.484 | 0.285 | 0.399 | 0.324 | 0.389 | 0.241 | 0.271 | 0.424 | 0.310 | 0.330 | 0.148 | 0.253 |  | 2.189 |
| ZJQZ | 0.258 | 0.194 | 0.167 | 0.331 | 0.476 | 0.406 | 0.244 | 0.320 | 0.268 | 0.284 | 0.167 | 0.224 | 0.313 | 0.273 | 0.235 | 0.062 | 0.147 | 0.102 |  |
